# Editorial: Developmental biology and endocrine research for a successful pregnancy

**DOI:** 10.3389/fendo.2024.1411864

**Published:** 2024-05-13

**Authors:** Jayonta Bhattacharjee, Alessandro Rolfo, Bellisa Freitas Barbosa, Kazuhiko Imakawa, Leonardo Ermini

**Affiliations:** ^1^ Department of Surgery and Obstetrics, Bangladesh Agricultural University, Mymensingh, Bangladesh; ^2^ Department of Surgical Sciences, School of Medicine, University of Turin, Turin, Italy; ^3^ Laboratory of Immunophysiology of Reproduction, Institute of Biomedical Science, Federal University of Uberlândia, Uberlândia, Brazil; ^4^ Research Institute of Agriculture, Tokai University, Kumamoto, Japan; ^5^ Department of Life Sciences, University of Siena, Siena, Italy

**Keywords:** pregnancy, placenta, trophoblast, developmental-biology, endocrine-research

Pregnancy is central to mammals’ lives, growth, and development. A healthy pregnancy is not only a prerequisite for a species’ survival and existence but also crucial for the growth and development of livestock and agriculture sectors, such as dairy and beef animals. Pregnancy failure and fertility have long been studied in developmental biology and endocrinology, and new inventions and technologies are welcome in this field.

Many factors, including endocrine disruptors and sedentary lifestyles, are becoming more prevalent in modern culture, limiting and impeding pregnancy success. Thus, reproductive development, endocrinology, and infectious mechanisms in pregnancy must be understood. Proper knowledge of reproductive development and endocrinological techniques will enable us to develop strategies to mitigate the pathologies and factors affecting a successful pregnancy.

Multiple reproductive organs regulate developmental biology. Starting from external and accessory reproductive organs, the uterus, oviduct, ovaries, and their health and optimum function are prerequisites for a successful pregnancy. Along with the reproductive organs, many other factors also regulate pregnancy success. Lifestyle factors, such as physical activity, good nutrition, and exposure to endocrine disruptors, influence reproductive and developmental outcomes. Knowing about all disorders and conditions affecting reproductive development and their management is crucial.

The objective of the Research Topic *“Developmental Biology and Endocrine Research for a Successful Pregnancy”* was to bring together and highlight the original research and reviews on the recent advancements in the work for successful pregnancy. This Research Topic includes a diverse range of studies, including seven original research articles, six reviews, and two retrospective cohort studies.

A successful pregnancy concerns us all. Knowing the phases and disorders of female reproductive stages is crucial. Liu et al reviewed the role of oxytocin (OT) in the health and disease of women. They explained how OT controls the reproductive cycle and pregnancy’s physiological phases. OT helps GnRH release, ovulation, lactation, fetus expulsion, milk ejection, and maternal behavior. This review also discusses how OT affects maternal depression and hypogalactia. Endocrine-Disrupting Chemicals (EDCs) and physiological endocrine or hormonal systems are of concern today. EDCs originate from both natural sources, such as plants, and industrial/artificial sources. Pool et al. examined how phytoestrogens affect sheep reproduction. They discussed the way estrogenic pasture impairs sheep’s reproduction. The relevance of estrogenic herbs on reproduction and pregnancy problems, including dystocia, stillborn lambs, and uterine prolapse after parturition (1–4), was stressed in their review. Endocrine disruptive substances’ effects on specific *in-utero* development periods are poorly understood. If phytoestrogens breach the blood-placenta barrier and enter fetal tissue (5, 6), Pool et al. warn that estrogenic substances may affect gestational outcomes *in utero*. Although the long-term effect of *in-utero* exposure to phytoestrogens have not yet been established, neonatal exposure has been documented to cause precocious vaginal opening (7), ovarian follicle atresia (8), increased uterine fluid content (9), and hyperplasia of the endometrium (9–11) in rodents. Basak et al. also discussed that maternal endocrine balance is crucial for pregnancy. EDCs in daily life can impact pregnancy and outcomes if they exceed permissible concentrations, affecting implantation, placental development, fetal organ development, and epigenetics (12–16). Therefore, EDC awareness is crucial for reproduction, fertility, and pregnancy success. Basak et al review the effects of EDCs, specifically bisphenols and phthalates, on fetoplacental growth and pregnancy outcomes.

Maternal obesity, gestational diabetes, preeclampsia, and cardiomyopathy are prominent pregnancy-related disorders. Exercise is safe and useful during pregnancy. Regular aerobic, anaerobic, and circular activities help pregnant humans and animals (17–19). Pahlavani reviewed the role of exercise in combating pregnancy complications, focusing on apelin in exercise-induced pregnancy problem protection. Pahlavani‘s review sheds light on the molecular mechanisms of exercise, including the APLN/APJ system, which has been extensively studied across various body systems and species (20–25). Shokrollahi et al. found that buffalo ovarian follicles regulate the ALPN/APJ system, affecting buffalo fertility. They also highlighted the role of adipokines in endocrine control and steroidogenesis, suggesting that IGF1 or FSH-based involvement could aid in therapeutic ALPN/APJ system use.

The placenta and trophoblast are crucial for pregnancy establishment and maintenance, but abnormalities in the trophoblast and its protein and gene functions can lead to pregnancy termination. S100P is one of them, which was first purified and characterized from the placenta (26–28). Zhou et al. found that S100P positively regulates trophoblast syncytialization during the earlier stages of pregnancy establishment through the regulation of YAP1 protein and is present in many other tissues as well (29, 30). The results from Zhou et al. showed lower S100P as a poor pregnancy outcome marker for humans. Another peptide and its receptor, kisspeptin, have an established role in reproduction as a whole (31–33). It is expressed in a wider range of tissue along with the placenta. Researchers depicted its role in pregnancy (34, 35). Tsoutsouki et al have explored kisspeptin levels as potential pregnancy complication markers. They suggest kisspeptin levels could be useful in early pregnancy losses alongside beta-HCG measurement. Kisspeptin levels have been found to be variable during preeclampsia, suggesting a need for further study to ensure pregnancy health and success. In this Research Topic, Zhou et al. studied the role of VTCN1 at the human maternal-fetal interface. VTCN1 (B7-H4)’s expression in the first week’s villous trophoblast was recently described (36). As VTCN1 has several functions and roles in immune homeostasis, its immunological involvement at the maternal-fetal interface could also be an important factor. Zhou et al. explained VTCN1 as an important regulator of trophoblast syncytialization and invasiveness during early placentation. Hence, VTCN1 could also be involved with abnormal placentation and diseases associated with abnormal placentation during pregnancy. The normal function of the placenta is also dependent on the ubiquitously expressed renin-angiotensin system (RAS) (37). The components of RAS, Angiotensin II (Ang II), and Angiotensin (1-7) (Ang-(1-7)) contributed to the normal physiological function of the reproductive processes such as follicular growth and development and the function of the placenta. Liu et al. summarize the localization and role of Ang II and Ang-(1-7) in the female reproductive system. A concreted understanding of the RAS and involvement of Ang II and Ang-(1-7) may aid in the understanding and maintenance of a healthy pregnancy. Whenever we consider the placental abnormalities, Kong et al., with a retrospective cohort study, found that IVF and maternal age are related to placental abnormalities. Extensive research is needed to determine if there is a connection between biological and molecular mechanisms causing placental pathological conditions in IVF and aged human pregnancies. Along with human studies, Bai et al. studied the insight into bovine pregnancy establishment and the role of the stromal protein PGE2 in bovines. Along with IFN tau, PGE2 from endometrial stromal cells helps maintain pregnancy with its luteoprotective function. Bai et al. demonstrated the molecular mechanism of how PGE2 helps in pregnancy maintenance. Bai et al. identified the PGE2-mediated factors viz. NFIL3 and CEBPA expression, might help in early-stage pregnancy establishment.

Polycystic ovary syndrome (PCOS) affects 6 to 15% of women of reproductive age (38). PCOS is also associated with miscarriage, gestational diabetes mellitus, hypertensive disorders of pregnancy, preterm delivery, and the birth of small-for-gestational-age (SGA) infants. Considering mRNA levels, Ren et al. stated that the PNA mouse is the best animal model for studying PCOS. Although a high percentage of women are affected by PCOS, its screening is not yet straightforward. Therefore, biomarkers could be of paramount importance here. Earlier studies highlighted that follicular microenvironments are related to PCOS (39). The pilot study by Ding et al., with the aim of searching for biomarkers of PCOS from follicular fluid, has successfully identified twenty-three lipid subclasses as potential biomarkers of PCOS in women. This study could help in developing diagnostic markers and an accurate and early screen of PCOS in women.

SGA and large-for-gestational-age (LGA) infants are always at greater risk of obstetrical and gynecological complications (40, 41). Much research has been conducted with regard to the transportation functions of the placenta. Maternal lipid viz, total cholesterol (TC), triglyceride (TG), low-density lipoprotein cholesterol (LDL-c), and high-density lipoprotein cholesterol (HDL-c) are taken up by the placenta and actively participate in maternal-fetal metabolism and development (42, 43). In their retrospective study, Zhu et al. found that a higher lipid profile is associated with higher birth weight in the first trimester. They also found an increased risk of (LGA) and macrosomia. Although it is not routinely practiced, Zhu et al. show that measuring pregnant women’s first trimester’s lipid profile is advisable.

However, comparatively less research has been conducted on the placenta, an endocrine organ. Placental-secreted hormones and growth factors directly contribute to fetal development and pregnancy maintenance (44–49). Lopez-Tello; Sferruzzi-Perri explored the placental endocrine function and identified placental hormones as key fetal growth and pregnancy maintenance regulators. In conclusion, it could be stated that this Research Topic combined research articles that could help researchers and scientists generate ideas for a sound and healthy pregnancy outcome.

## Author contributions

JB: Conceptualization, Writing – original draft, Writing – review & editing. AR: Writing – review & editing. BB: Writing – review & editing. KI: Writing – review & editing. LE: Writing – review & editing.
